# Morphology of muscle attachment sites in the modern human hand does not reflect muscle architecture

**DOI:** 10.1038/srep28353

**Published:** 2016-06-23

**Authors:** E. M. Williams-Hatala, K. G. Hatala, S. Hiles, K. N. Rabey

**Affiliations:** 1 Department of Biology, Chatham University, Woodland Road, Pittsburgh, PA 15232, USA; 2 Center for the Advanced Study of Human Paleobiology, The George Washington University, 800 22^nd^ St., NW, Suite 6000, Washington, DC 20052, USA; 3 Department of Human Evolution, Max Planck Institute for Evolutionary Anthropology, Deutscher Platz 6, D-04103 Leipzig, Germany; 4 Department of Anatomy, Midwestern University, 555 31^st^ St., Downers Grove, IL 60515, USA; 5 Department of Evolutionary Anthropology, Duke University, 104 Biological Sciences Building, Durham, NC 27708, USA

## Abstract

Muscle attachment sites (entheses) on dry bones are regularly used by paleontologists to infer soft tissue anatomy and to reconstruct behaviors of extinct organisms. This method is commonly applied to fossil hominin hand bones to assess their abilities to participate in Paleolithic stone tool behaviors. Little is known, however, about how or even whether muscle anatomy and activity regimes influence the morphologies of their entheses, especially in the hand. Using the opponens muscles from a sample of modern humans, we tested the hypothesis that aspects of hand muscle architecture that are known to be influenced by behavior correlate with the size and shape of their associated entheses. Results show no consistent relationships between these behaviorally-influenced aspects of muscle architecture and entheseal morphology. Consequently, it is likely premature to infer patterns of behavior, such as stone tool making in fossil hominins, from these same entheses.

Biologists and paleontologists frequently have scant data available to them from which to reconstruct the anatomy, physiology and behaviors of past populations. Consequently, fossils and artifacts are scoured for information on the lives of the individuals that left them behind. Entheses (attachment sites of soft tissue) are popular osteological features used to reconstruct soft tissue anatomy, and those reconstructions are regularly employed to infer behavior of past populations. Their use stems from the fact that some entheses tend to be overtly visible on bone, and are thought to reflect some aspects of the anatomy and physiology of the corresponding muscle (e.g., refs 1, 2, 3, 4, 5, 6). Due to their physical connections to muscles and tendons, and their high inter-individual variability in size and shape, entheses are often used as direct inferential bases to reconstruct muscle anatomy of extinct individuals, past behaviors and, at times, even the degree of participation in these behaviors (e.g., refs 7, 8, 9, 10, 11, 12, 13).

The advent of our ancestors’ participation in stone tool behaviors (i.e., tool use and production) is thought to demarcate an important adaptive shift and to have provided the impetus for other influential changes throughout human evolution^14^,^15^,^16^,^17^. Yet due to a sparse fossil record, discerning the exact origin(s) of hominin stone tool behaviors and the hominin taxa that made and used tools are difficult to pinpoint. The hypothetical direct relationship between entheseal morphology and muscle anatomy is routinely invoked to estimate the architecture of muscles heavily recruited during stone tool behaviors (e.g., refs 18, 19, 20, 21, 22, 23). Resulting muscle reconstructions have then been used to suggest tool behaviors in certain fossil hominins. Simultaneously, these assumptions (indirectly) confer a suite of cognitive and motor abilities to individuals or, often, entire species.

However, the anatomy, physiology and mechanical properties of tendon-bone interfaces are complex and the use of entheses as direct behavioral indicators fails to consider the multifactorial influences on entheseal morphologies. These influences include muscle function^1^,^24^,^25^,^26^ as well as enthesis type, biomechanics, and numerous extrinsic variables (e.g., age, sex, genetics, health variables) that are known to affect both entheseal morphology specifically and bone and muscle growth in general^2^,^3^,^4^,^27^,^28^,^29^. Reconstructions of anatomy and behavior based on the analysis of entheseal gross surface morphology likely oversimplify the relationship between the two. Given the nature of the many variables that influence the morphology of the tendon-bone junction, it is also likely that the dynamics governing entheseal morphology will vary across species and even across muscle locations within the same individual, irrespective of behavior^25^,^30^. If this is the case, then it may be impossible to use particular entheses as direct indicators of the architecture of associated muscles and certainly of stone tool behaviors, much less to do so across multiple fossil hominin taxa.

Here, we conducted the first direct assessment of the assumed relationships between muscle anatomy and entheseal morphology that are most commonly used to infer levels of participation in stone tool behaviors among fossil hominins: the entheseal markings of opponens pollicis (OP) and opponens digiti minimi (ODM) on the first and fifth metacarpals (MC), respectively. Working with the other thenar and hypothenar muscles, the opponens muscles participate in full and forceful opposition of the pads of the distal phalanges of the first and fifth digits. Opposition is regarded as a significant biomechanical component of stone tool behaviors^31^,^32^, which has led to the wide use of the OP and ODM entheses to reconstruct muscle anatomy and to infer tool behaviors in fossil hominins^18^,^33^,^34^.

## Results

**Figure 1:**
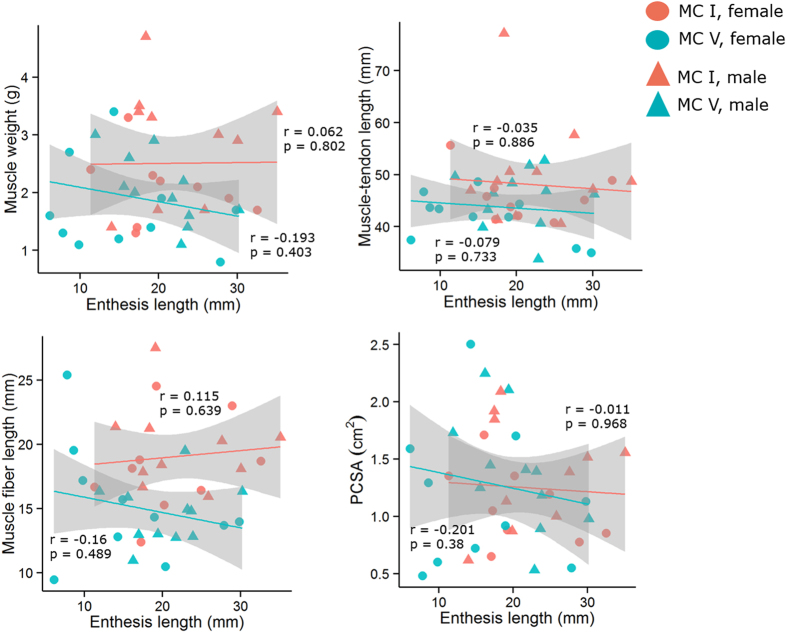
Correlations of entheseal length and muscle architectural variables. From left to right and top to bottom, plots show entheseal length versus muscle mass, muscle-tendon length (MTU), fiber length (L_f_), and physiological cross-sectional area (PCSA). Lines designate predicted linear regression fits, for visualization purposes only, and were calculated separately from first and fifth metacarpal data. The 95% confidence intervals of those linear regression fits are shown in grey.

**Figure 2:**
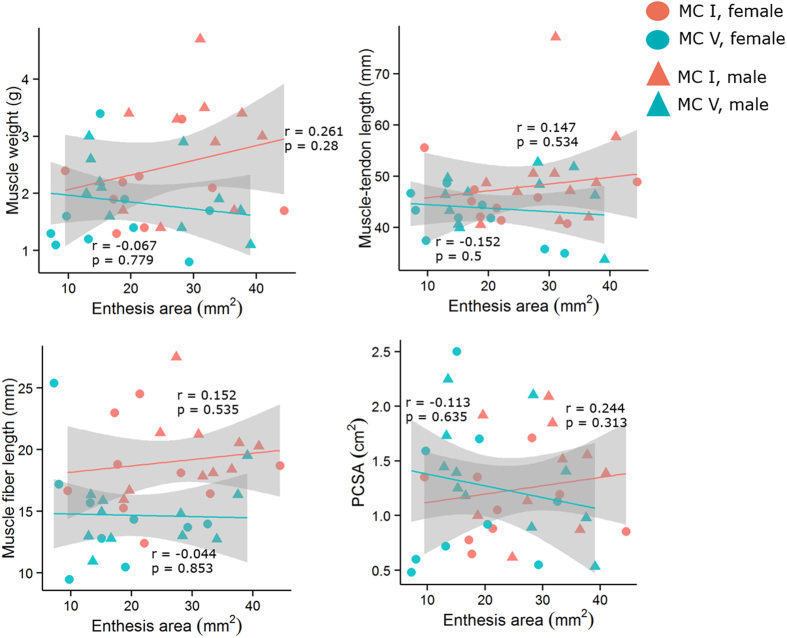
Correlations of entheseal area and muscle architectural variables. From left to right and top to bottom, plots show entheseal area versus muscle mass, muscle-tendon length (MTU), fiber length (L_f_), and physiological cross-sectional area (PCSA). Lines designate predicted linear regression fits, for visualization purposes only, and were calculated separately from first and fifth metacarpal data. The 95% confidence intervals of those linear regression fits are shown in grey.

**Figure 3:**
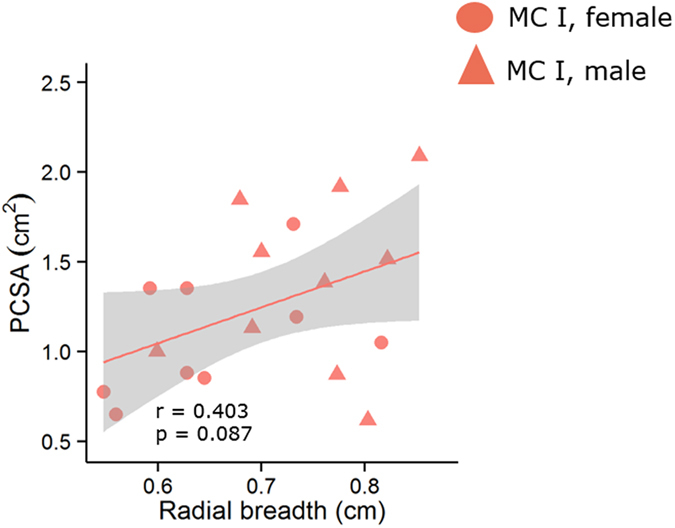
Relationship between radial breadth and PCSA. Line designates predicted linear regression fit, for visualization purposes only, and the 95% confidence interval of that fit is shown in grey.

Using 23 cadaveric modern humans (46 metacarpals), we tested whether any correlations existed between a series of functionally-influenced architectural variables describing the opponens muscles (muscle mass, muscle-tendon length [MTU], fiber length [L_f_] and physiological cross-sectional area [PCSA]) and their associated entheses (length, area, and radial breadth). All correlations between opponens muscle architectural variables and their associated entheseal surface morphology variables lacked statistical significance (P > 0.05), regardless of metacarpal and sex (Table 1 and Figs 1, 2, 3). Most correlation coefficients were extremely small and highly insignificant with the exception of the correlation between radial breadth and PCSA, which was noticeably stronger than the rest but still statistically insignificant (Table 1, Fig. 3).

## Discussion

Some scientists studying the osteological remains of past populations have made a practice of reconstructing muscle anatomy and behavioral patterns of skeletal or fossil individuals from the entheseal morphology preserved on the surface of dry bones. This method has always been questionable given the complexity of osteotendinous interfaces and the number of variables known to influence bone and muscle growth (e.g., refs 1, 2, 3, 4, 5, 6). With this history in mind, we tested the validity of the hypothesized relationship between muscle anatomy and entheseal morphology by focusing on two entheses (and their associated muscles of the hand) that are commonly used to infer tool behaviors in our fossil human ancestors. We considered four functionally influenced variables describing muscle and tendon anatomy (L_f_, MTU, PCSA, and mass) and three variables describing the associated entheses (length, area, and radial breadth). In all comparisons, correlations were very weak and statistically insignificant. These results provide evidence that the entheseal morphologies of the opponens muscles are not direct correlates that can be used to reconstruct the associated muscle architecture, and therefore, function. Consequently, these data further argue against the practice of inferring behavior, stone tool or otherwise, from entheseal morphology preserved on the surfaces of the first and fifth metacarpals of fossils.

Entheses are complex, and numerous variables acting at the enthesis influence its morphology. The structural complexity of entheses is apparent at the most basic classification level: their categorization as either fibrous and fibrocartilaginous entheses^35^. In general, these types are differentiated grossly by their observed attachment location (diaphysis versus epiphysis or apophysis, respectively) and histologically by the composition of the tissues at the tendon-bone interface^2^. However, many limb muscles that attach via fibrocartilaginous entheses have zones of dense fibrous connective tissue (i.e., a fibrous enthesis) along the superficial part of the enthesis^2^. Benjamin *et al.*^2^ attribute such mixed entheses to differing mechanics between the enthesis types. In other words, fibrocartilaginous and fibrous entheses respond to stress and strain differently, and further, they transfer such forces onto underlying bone differently. Unfortunately the specifics of how either type of enthesis interacts with bone are only partially understood^2^,^28^,^36^,^37^, and even less is known regarding the interaction of bone with mixed entheses.

Regarding their different biomechanics, fibrocartilaginous entheses generally have relatively small and distinct attachment sites, resulting in a high concentration of stress of the underlying bone. Fibrous entheses, on the other hand, tend to attach over a relatively larger area of bone, either via the periosteum or by inserting directly into the bone^35^. The relatively larger insertion area distributes stresses over a larger area, decreasing the magnitude of the force acting at the enthesis. In light of these simple mechanical differences, the use of a single observational method to analyze entheses throughout the body^7^ is likely insufficient to account for the ways in which the different enthesis types respond to stress in different ways at different locations^28^. Steps to account for the impacts of these differences are rarely taken.

Irrespective of entheseal type and perhaps more problematic toward the practice of reconstructing soft tissue from hard tissue remains is the fact that tendons exhibit non-uniform strain patterns near their insertion site and initial sites of tendinopathy and enthesopathy do not correspond to primary or initial areas of force transfer^38^. In addition to highlighting the complex nature of osteotendinous interface, this suggests that associated diseases may arise from under-use rather than over-use. Some (e.g., refs 1,6,39) have argued that entheses may develop as a protective response to injury, so muscle contractions (forceful or lack thereof) may not impact entheseal morphology until a pathological state is reached. Thus, areas exhibiting exaggerated entheses or enthesopathies do not necessarily reflect a habitual pattern of heavy muscle recruitment.

Another practical problem with qualitative enthesis analysis is that in many instances multiple soft tissue structures (tendons, ligaments, and connective tissues) insert along or close to the same area^40^. Thus a given enthesis may experience (and potentially reflect) forces from multiple structures rather than a single muscle. Furthermore, previous paleontological and bioarchaeological studies assume that all species modify their entheses in a similar fashion and at the same rate. If one species has a quicker morphological transformation of its entheses in response to similar muscle contractions than a second species, the first is likely to present wider variations of morphologies, while the latter will be more homogeneous^41^. This causes further problems when using entheses to draw interspecies comparisons of behavior.

While it is premature to extend our results across the human body, or to other mammalian taxa, a consistent pattern has begun to emerge in which multiple direct tests have failed to support assumed relationships between enthesis morphology, location, or even presence of the muscle with muscle anatomy or behavior^39^,^42^,^43^. For example, two studies conducted on human cadavers found that in some cases a presumed enthesis was present in areas where the associated muscle did not actually attach^43^ or a presumed enthesis existed when the associated muscle was entirely absent^42^. Similarly, experimental studies with mice and sheep found no differences in enthesis morphology between groups that was subject to regular exercise and groups with limited exercise regimes^1^,^39^. Our results, alongside these similar findings, argue against the practice of reconstructing anatomy or daily behavior in fossil taxa based on any surface entheses, until concrete experimental evidence can be shown to support such a relationship.

## Materials and Methods

### Sample

Soft tissues were obtained from 23 adult humans from Duke University School of Medicine, including 12 males and 11 females (average age: 77.9 ± 12 years). All cadavers were fixed according to standard preservation protocol used by Duke University. All donors signed informed consent forms to formally donate tissues to Duke University for education and research purposes. Duke University School of Medicine approved all experimental methods and all research was conducted in accordance with the umbrella IRB of Duke University School of Medicine.

### Muscle dissection and variables

The sample was comprised of the opponens pollicis (OP) and the opponens digiti minimi (ODM) muscles from the first and fifth metacarpals, respectively, of the right hand. To begin dissection, the skin and superficial fascia across the palmar surface of the hand, the first and fifth digits, and the forearm was removed. Muscles and tendons of interest were differentiated, photographed, and measured *in situ* using digital calipers prior to muscle removal. Muscle belly length (L_b_), total tendon length (L_T_) and the full muscle-tendon unit (MTU) were measured to the nearest 0.1 millimeter. The muscle belly length (L_b_) was defined as the length of the muscle fasciculus between the proximal and distal myotendinous junctions^44^. The presence of proximal and distal tendons varied significantly on an intra- and inter-individual basis. Therefore, the total tendon length (L_T_) was defined as the sum of the lengths of the proximal and/or distal muscle tendons^44^. And the muscle-tendon unit (MTU) was defined as the sum of L_b_ and L_T_. Following measurement, the muscles and their associated tendon(s) were removed in a manner that preserved as much of the tendon and muscle tissue as possible, and cleaned of excess tissues. Each muscle was stored in a separate storage container in a 10% formalin solution to maintain preservation and fixation. Remaining superficial soft tissues and fibers were removed from the first and fifth digits, and the digits were detached from the hand at the carpometacarpal joints and stored in a 10% formalin solution.

Prior to further analysis, muscles were removed from the formalin and partially dried to remove excess liquids. Each muscle mass was measured to the nearest 0.1 gram using a digital scale. The muscle was then pinned to a vinyl dissecting pad and three longitudinal incisions were made along the line of action (one superficial, one deep, and one on the reverse side of the muscle on the cleanest surface available). Two measurements were taken for each incision: (a), fiber length (L_f_: for a given muscle fiber, the distance between the central tendon of origin to the distal tendon of insertion) and (b), the perpendicular distance between the central tendon to the tendon of insertion (see Fig. 4.2)^44^. Each measurement was taken three times and the mean was used to calculate the angle of pennation (Θ = arcsin a/L_f_). Physiological cross-sectional area (PCSA) was defined as:


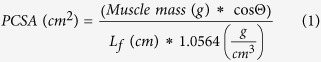


where 1.0564 g/cm^3^ is an estimate of muscle density value^45^. Fiber architecture refers to the arrangement of muscle fibers relative to the force-generating axis of the muscle^24^,^46^,^47^,^48^. Fiber length and PCSA are especially important determinants of muscle function^1^. The length of a fiber has been shown to be proportional to a muscle’s maximum excursion, and by extension, velocity of contraction^49^,^50^; while PCSA has been shown to be proportional to the maximum force a muscle can generate^51^.

### Skeletonization and entheseal measurements

Following dissection, the digits were placed in a 5% Terg-A-Zyme (Alconox, NY) bath to further remove soft tissues. The bath was changed every other day for one month. After this period of time, the digits were boiled in individual Terg-A-Zyme baths for two hours and wiped clean of remaining soft tissues. Once the bones dried to completion, the length of the opponens ridge (i.e., the insertion point for the opponens muscles) was measured on each metacarpal using digital calipers. Each ridge was measured three times and the mean was used for the correlation analyses.

**Figure 4:**
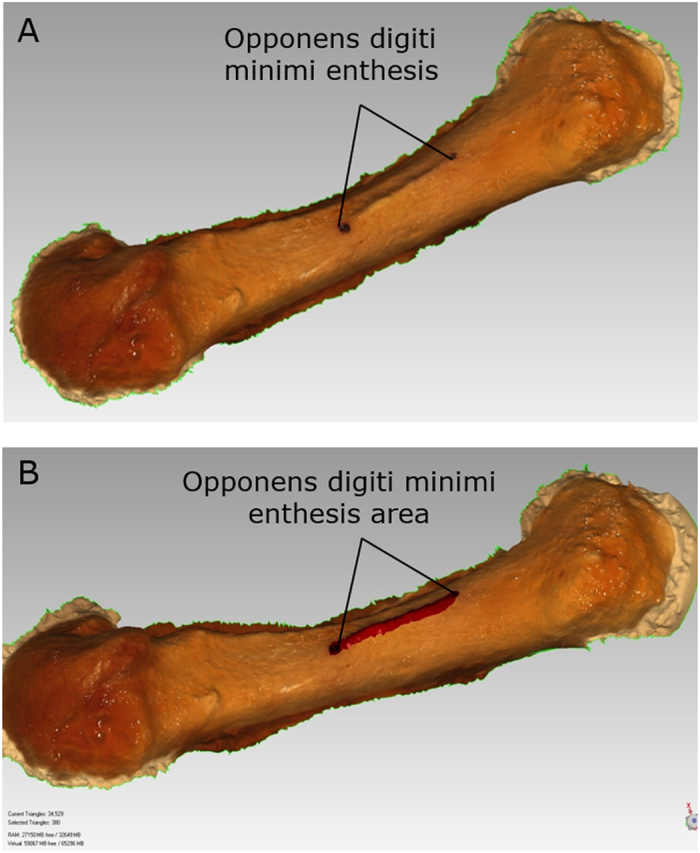
3-dimensional polygonal models of a female fifth metacarpal showing the enthesis of opponens digiti minimi (**A**) and the same region selected in red to calculate entheseal area (**B**).

Area of each enthesis was measured on three-dimensional digital models of each bone, as virtual surface area measurements could incorporate the complex three-dimensional topography of each enthesis. Three-dimensional models of each bone were created by photogrammetry. Approximately 20 photos of each bone were taken from various angles using a Canon 5D Mark III 22.3 megapixel camera fitted with a 50 mm macro lens. Photos were loaded into Agisoft PhotoScan Professional software (Agisoft, LLC, St. Petersburg, Russia) such that 3-dimensional polygonal models could be rendered from the images using structure from motion algorithms. The maximum length of each enthesis was demarcated on each metacarpal in order to scale the 3-D models for analysis. Following their creation, polygonal models were exported and loaded into Geomagic Studio 2014 (3D Systems Inc., Rock Hill, SC) for geometric analysis. Entheseal surface area was determined based on a combination of visual observations of the bone of interest and of the resulting models (Fig. 4B). The area of the enthesis was selected and its 3-D surface area was measured using the measurement tool available within the Geomagic software. A sample of ten entheses (5 OP and 5 ODM) were measured three times each by KGH and EMWH. Intra- and inter-observer variation were calculated and determined to be negligible relative to the size of the measured entheses (average inter-observer error = 0.98 mm^2^ or 4.0% of measurement of interest; average intra-observer error = 0.69 mm^2^ or 2.6% of measurement of interest). Given the negligible differences, the remaining 36 entheses were divided and measured by either KGH or EMWH.

**Figure 5:**
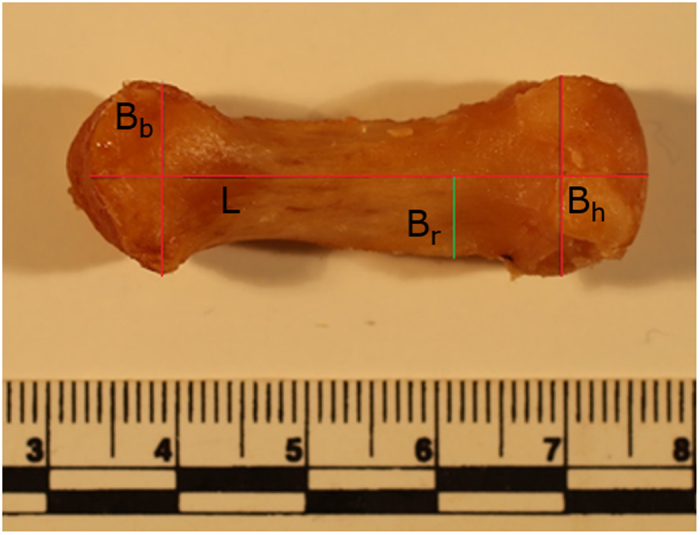
Radial breadth. Measurements taken to quantify radial breadth following Maki and Trinkaus^52^. Maximum head breadth (B_h_), and maximum base breadth (B_b_) were measured on each metacarpal. Maximum length (ML) is defined as the line bisecting B_h_ and B_b_ along the long proximal–to-distal axis of the metacarpal. Radial breadth (B_r_) was defined as the line from the point at 65% of ML to the lateral margin of the metacarpal as represented in the palmar photograph.

Maki and Trinkaus^52^ used the radial deviation of the opponens ridge present on pollical metacarpals (i.e., radial breadth) to infer PCSA as a proxy for the moment arm and effective mechanical advantage of opponens pollicis muscles in Neanderthals. A muscle’s effective mechanical advantage is a function of physiological cross section area and muscle mass so, in other words, they argued that radial breadth reflects in some way muscle mass and/or PCSA. However, the relationship between this measurement of the opponens pollicis enthesis and the anatomy of the muscle itself had not been specifically examined until now. Here, radial breadth was measured from scaled photographs of the pollical metacarpals following the method described in Maki and Trinkaus^52^ (Fig. 5). A photograph of the palmar view of each metacarpal was selected and loaded into ImageJ^53^. Maximum length (ML), maximum head breadth (B_h_), and maximum base breadth (B_b_) were measured on each metacarpal. Maximum length was defined as the line bisecting B_h_ and B_b_, running along the long proximal –to-distal axis of the metacarpal^52^. Radial breadth (B_r_) was measured from the point at 65% of ML to the lateral margin of the metacarpal as represented in the palmar photograph.

### Statistical Analysis

Data and analyses were separated by metacarpal and by sex. Spearman’s rank correlations were used to test the relationship between three variables describing the opponens muscles (MTU, PCSA, L_f_ and mass) and variables describing their associated enthesis (length, area, and radial breadth). Post-hoc tests of significance were run on each correlation. All analyses were conducted using custom-written scripts in the R programming language and environment^54^.

### Code availability

Computer code used to conduct statistical analyses of the data is available upon request from the lead author.

## Additional Information

**How to cite this article**: Williams-Hatala, E. M. *et al*. Morphology of muscle attachment sites in the modern human hand does not reflect muscle architecture. *Sci. Rep.* **6**, 28353; doi: 10.1038/srep28353 (2016).

## Figures and Tables

**Table 1:** Correlations between entheseal and muscular variables, MC1 and MC5.

MC1			MC5		
Correlation	r	p-value	Correlation	r	p-value
Enthesis length and muscle mass	0.062	0.802	Enthesis length and muscle mass	−0.193	0.403
Enthesis length and MTL	−0.035	0.886	Enthesis length and MTL	−0.079	0.733
Enthesis length and fiber length	0.115	0.639	Enthesis length and fiber length	−0.16	0.489
Enthesis length and PCSA	−0.011	0.968	Enthesis length and PCSA	−0.201	0.38
Enthesis area and muscle mass	0.261	0.28	Enthesis area and muscle mass	−0.067	0.779
Enthesis area and MTL	0.147	0.534	Enthesis area and MTL	−0.152	0.5
Enthesis area and fiber length	0.152	0.535	Enthesis area and fiber length	−0.044	0.853
Enthesis area and PCSA	0.244	0.313	Enthesis area and PCSA	−0.113	0.635
Radial breadth and PCSA	0.403	0.087			

MTL: muscle-tendon length. PCSA: physiological cross-sectional area.
